# Expression and Functional Identification of *SPL6/7/9* Genes under Drought Stress in Sugarbeet Seedlings

**DOI:** 10.3390/ijms25168989

**Published:** 2024-08-18

**Authors:** Hui Wang, Shengyi Zhu, Chao Yang, Deyong Zeng, Chengfei Luo, Cuihong Dai, Dayou Cheng, Xiaohong Lv

**Affiliations:** 1 School of Chemistry and Chemical Engineering, Harbin Institute of Technology, Harbin 150001, China; wang_hui@hit.edu.cn (H.W.); zhushengyihit@163.com (S.Z.); cfluo7375@hit.edu.cn (C.L.); 2 School of Astronautics, Harbin Institute of Technology, Harbin 150001, China; 15201702938@163.com; 3 School of Medicine and Health, Harbin Institute of Technology, Harbin 150001, China; 18b925086@stu.hit.edu.cn; 4 Heilongjiang Academy of Forestry, Harbin 150001, China; 18645105533@163.com

**Keywords:** *Beta vulgaris* L., drought stress, SPL, plant stress resistance

## Abstract

Sugar beet is a significant sugar crop in China, primarily cultivated in arid regions of the north. However, drought often affects sugar beet cultivation, leading to reduced yield and quality. Therefore, understanding the impact of drought on sugar beets and studying their drought tolerance is crucial. Previous research has examined the role of SPL (*SQUAMOSA* promoter-binding protein-like) transcription factors in plant stress response; however, the precise contribution of SPLs to the drought stress response in sugar beets has yet to be elucidated. In this study, we identified and examined the *BvSPL6*, *BvSPL7*, and *BvSPL9* genes in sugar beets, investigating their performance during the seedling stage under drought stress. We explored their drought resistance characteristics using bioinformatics, quantitative analysis, physiological experiments, and molecular biology experiments. Drought stress and rehydration treatments were applied to sugar beet seedlings, and the expression levels of *BvSPL6*, *BvSPL7*, and *BvSPL9* genes in leaves were quantitatively analyzed at 11 different time points to evaluate sugar beets’ response and tolerance to drought stress. Results indicated that the expression level of the *BvSPL6/9* genes in leaves was upregulated during the mid-stage of drought stress and downregulated during the early and late stages. Additionally, the expression level of the *BvSPL7* gene gradually increased with the duration of drought stress. Through analyzing changes in physiological indicators during different time periods of drought stress and rehydration treatment, we speculated that the regulation of *BvSPL6/7/9* genes is associated with sugar beet drought resistance and their participation in drought stress response. Furthermore, we cloned the CDS sequences of *BvSPL6*, *BvSPL7*, and *BvSPL9* genes from sugar beets and conducted sequence alignment with the database to validate the results. Subsequently, we constructed overexpression vectors, named 35S::*BvSPL6*, 35S::*BvSPL7*, and 35S::*BvSPL9*, and introduced them into sugar beets using *Agrobacterium*-mediated methods. Real-time fluorescence quantitative analysis revealed that the expression levels of *BvSPL6*/7/*9* genes in transgenic sugar beets increased by 40% to 80%. The drought resistance of transgenic sugar beets was significantly enhanced compared with the control group.

## 1. Introduction

Beet (*Beta vulgaris* L.), a biennial herbaceous plant belonging to the Amaranthaceae family and the genus Beta, is classified into various categories, including edible sugar beet, fodder sugar beet, and sugar beet, each possessing significant application value [1]. Drought represents one of the most severe and widespread natural disasters globally. In China alone, approximately 20.9 million hectares of arable land are affected by drought, making it a primary factor that impacts agricultural production [2]. The adverse effects of drought can permeate the entire sugar beet growing season, significantly compromising both the quality and yield of sugar beets, resulting in an estimated economic loss of one hundred billion US dollars to global agriculture [3].

The SPL transcription factor represents a plant-specific gene family primarily responsible for regulating the plant growth cycle, stress resistance, and other essential traits [4]. Research has shown that plants can adapt to environmental stress by modulating the SPL transcription factor gene via *miR156* under various stress conditions [5]. SPL proteins exhibit a remarkable versatility in their conserved residues. Nevertheless, these proteins share a highly conserved region consisting of 76 amino acids, known as the *SQUA* promoter-binding proteins (SBP) domain [6]. This domain features two zinc finger binding sites and a nuclear localization signal. The first identified SPL proteins, AmSBP1 and AmSBP2, originate from the same lineage as the *Antirrhinum majus AP1* gene, which interacts with the promoter of early flowering in plants through the *SQUAMOSA* identity gene [7]. Fine-tuning of *SPL* gene expression might be a useful strategy for crop improvement. For instance, under repeated heat stress, the *SPL2* and *SPL11* genes exhibit significant negative regulation [8]. In the context of drought stress, the *SPL6* gene in alfalfa demonstrates robust positive effects under drought conditions [9]. In rice, *SPL6* directly interacts with the inositol-requiring enzyme 1 (IRE1) promoter, influencing IRE1 transcriptional activation through patterning and repression [10]. *SPL7* and its homolog are highly conserved in plants as steady copper adjustment factors, regulating copper balance by modulating abscisic acid (ABA) accumulation. Increased ABA accumulation inhibits plant growth, enhancing plant tolerance to drought. The *SPL9* gene plays diverse roles in plant developmental processes and responses to biotic stresses [11,12]. The *SPL9* gene has been found to be widely involved in many developmental processes and responses to biotic stresses in plants. Drought-induced suppression of *SPL9* expression in cassava affects drought resistance primarily by modulating secondary metabolites and jasmonic acid (JA) signaling [13]. Additionally, *SPL9* regulates anthocyanin biosynthesis in alfalfa, enhancing drought tolerance [14]. G. Xue et al. identify eight members of the sugar beet *SPL* gene family. SPL transcription factors play an important role in regulating plant growth and development and abiotic stress responses and may be involved in the regulation of root expansion and sugar accumulation in sugar beets [15]. These findings underscore the critical role of plant *SPL* genes in stress adaptation, although limited research exists on the involvement of *SPL* genes in sugar beet responses to drought stress.

Drought stress significantly impacts the biochemical characteristics of sugar beets, enhancing antioxidant enzyme activity while reducing the generation of reactive oxygen species (ROS), which is crucial for improving tolerance to salt stress [16]. Proline (PRO) is an amino acid that accumulates in plants under stress conditions such as drought, salinity, and low temperatures, serving essential roles in osmoregulation and cellular protection against oxidative damage. Peroxidase (POD), an important antioxidant enzyme, detoxifies ROS, including hydrogen peroxide, thereby mitigating oxidative damage and preserving cellular membrane integrity, making its activity a reliable indicator of stress tolerance. Superoxide dismutase (SOD) catalyzes the conversion of superoxide radicals into hydrogen peroxide and oxygen, playing a pivotal role in reducing ROS accumulation and protecting cells from oxidative injury. Catalase (CAT) complements SOD by decomposing hydrogen peroxide, further alleviating oxidative stress and safeguarding cellular macromolecules. Malondialdehyde (MDA), a byproduct of lipid peroxidation, serves as a biomarker for cell membrane damage, with its accumulation reflecting the integrity of plant membranes and overall stress tolerance. Additionally, chlorophyll content plays multiple roles in plant stress resistance, including contributions to photosynthesis, antioxidant capacity, and water use efficiency. By enhancing chlorophyll synthesis or maintaining its stability, plants can respond more effectively to environmental stress. Chlorophyll content plays multiple roles in plant stress resistance, including photosynthesis, antioxidant capacity, usage efficiency of water, and so on. By improving chlorophyll synthesis or maintaining its stability, plants can respond more effectively to environmental stress. Specifically, drought stress leads to a marked decrease in POD activity, while CAT activity in leaves is significantly upregulated [17]. In response to drought conditions, sugar beets rapidly synthesize osmotic regulatory substances to adapt to the arid environment, with PRO and MDA serving as the primary regulatory compounds. Notably, drought stress significantly increases PRO content in leaves while decreasing MDA levels [18]. Furthermore, drought stress induces alterations in primary metabolism; studies have shown that to maintain high yields, there must be both similarity and specificity in metabolic regulation between leaves and roots after a brief period of drought. This underscores the robust drought resistance of sugar beets, as rehydration prior to permanent wilting has been found to restore root vitality and ensure normal growth [19].

Currently, research on drought resistance in sugar beets primarily focuses on organizational and cellular levels, with an increasing emphasis on elucidating resistance mechanisms at the molecular level as a key area for future investigation. Analyzing the expression patterns of *SPL* genes in response to drought stress in sugar beet leaves establishes a foundation for further exploration of the stress resistance functions of these genes. Constructing an *SPL* genes overexpression vector and transforming sugar beets to validate the role of *SPL* in drought stress response is crucial for enhancing stress resilience in sugar beets. This approach offers new insights and strategies for developing drought-resistant sugar beets and other crops. In this study, we investigated the expression of the *BvSPL6*, *BvSPL7*, and *BvSPL9* genes in the sugar beet variety DF-2 under 72 h drought stress and subsequent 6 d rehydration at various time points through quantitative analysis. The expression vectors for *BvSPL6*, *BvSPL7*, and *BvSPL9* were successfully introduced into sugar beets via *Agrobacterium tumefaciens* transformation. Our findings indicated that sugar beets overexpressing *BvSPL6*, *BvSPL7*, and *BvSPL9* exhibited improved drought resistance.

## 2. Results

### 2.1. Analysis of BvSPL6/7/9 Genes Expression in Sugarbeet under Drought Stress

**Figure 1:**
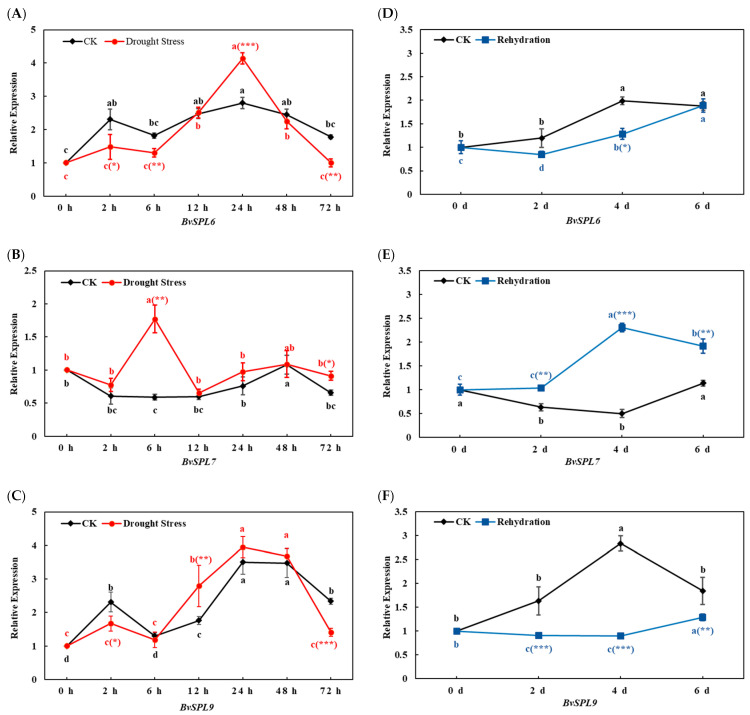
Analysis of *BvSPL6/7/9* gene expression in sugar beets under drought stress and rehydration treatment. Expression analysis of (**A**) *BvSPL6*, (**B**) *BvSPL7*, and (**C**) *BvSPL9* in sugar beets under drought stress. Expression analysis of (**D**) *BvSPL6*, (**E**) *BvSPL7*, and (**F**) *BvSPL9* in sugar beets under rehydration. The error line represents the standard deviation of three biological repetitions. Letter labeling and asterisk labeling are used to analyze, respectively, the intra-group and inter-group data significant differences. There is no significant difference among the same letter groups (*p* > 0.05), but there are significant differences among different letter groups (*p* < 0.05). *, **, and *** represent significant differences at *p* < 0.05, 0.01, and 0.001 levels between the CK (without drought treatment) group and the treatment groups at the same time point.

Fluorescence quantitative PCR technology was employed to assess the expression changes of the *BvSPL6*, *BvSPL7*, and *BvSPL9* genes in the leaves of sugar beet seedlings collected at seven time points during a 72 h drought stress treatment (Figure 1A–C). Additionally, the expression changes of *BvSPL6*, *BvSPL7*, and *BvSPL9* genes were analyzed every 2 days during a 6 d rehydration treatment (Figure 1D–F).

The expression of *BvSPL6* showed an obvious pattern in response to drought stress. It was initially lower in 0–6 h, increased significantly in 6–24 h, and then began to decrease until 72 h and then significantly lower within 48–72 h. In contrast, *BvSPL9* expression was lower in the early and late stages of drought stress than that in the CK group but significantly increased at 6 h, peaked at 24 h, and began to decline rapidly at 48 h. The expression of *BvSPL7* was consistently higher than that of the CK group during the whole 0–72 h period. It is worth noting that there is a rapid rise and a sharp decline in 2–12 h, with a maximum at 6 h. The inference is that the regulatory factors are complex, leading to regulation differences.

In the comparison of *BvSPL6*, *BvSPL7*, and *BvSPL9* expression between the CK group and the rehydration treatment group, it was observed that the expression levels of *BvSPL6* and *BvSPL9* in the first 6 days after rehydration treatment were lower than those in the CK group, and the expression level of *BvSPL7* was significantly higher than that of the CK group. Further analysis of the rehydration group data at different time points showed that the expression of *BvSPL6* was significantly upregulated to the highest level from 2–6 d and reached a 1.92-times increment on the 6th day, suggesting that the 6th day is the critical time point for *BvSPL6* to respond to water recovery. Similarly, *BvSPL9* was significantly upregulated from 4–6 d, reaching 1.28 times upregulation by 6 d. The expression level of *BvSPL7* increased significantly from 2 d to 4 d and reached 2.32-fold upregulation on the 4th day, suggesting that the 4th day is the key point for *BvSPL7* to respond to rehydration.

These results suggested that rewatering could effectively promote the recovery of sugar beets after 72 h of drought stress, and *BvSPL6/9* responded to rewatering by downregulating expression, while *BvSPL7* promoted recovery by upregulating expression. This indicated that the sugar beet had a low degree of irreversible damage under drought stress and had the ability to recover normal growth by rewatering.

### 2.2. Changes in Physiological Indicators of Sugar Beets under Drought Stress

**Figure 2:**
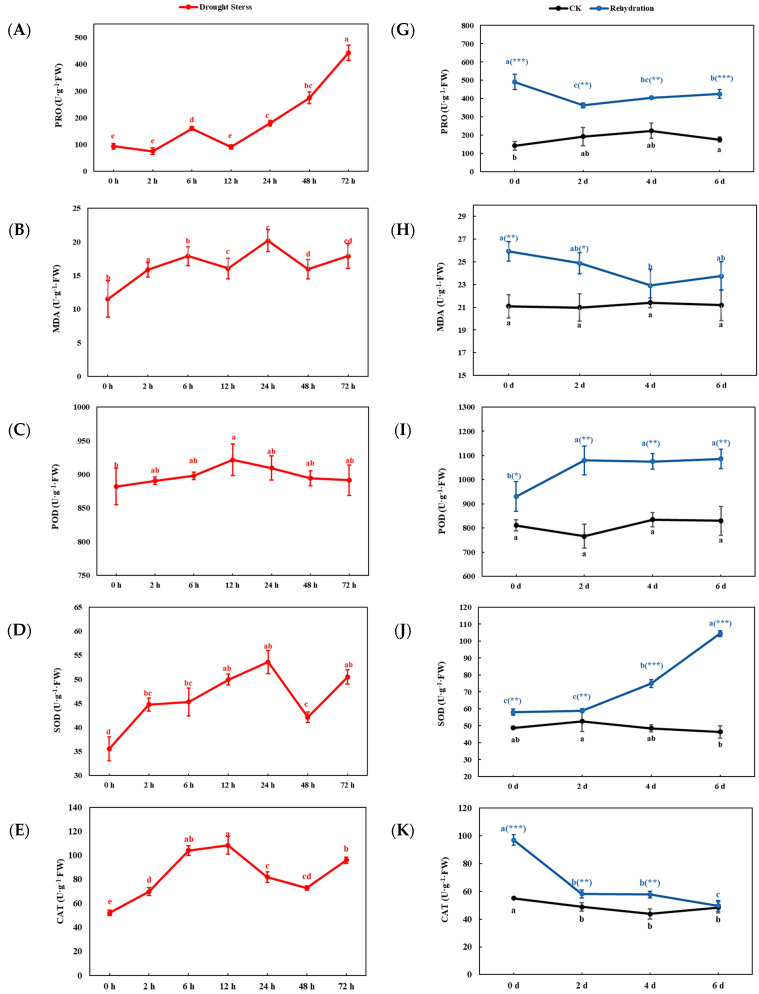

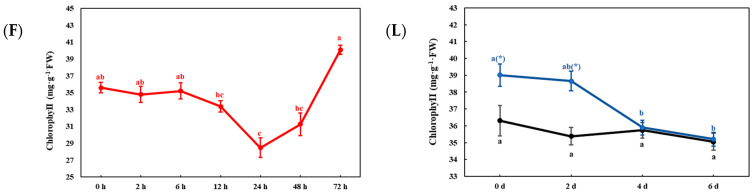
Effect of drought stress and rehydration treatment on PRO, MDA, POD, SOD, CAT, and chlorophyll content in beet leaves. (**A**–**F**) The PRO, MDA, POD, SOD, CAT, and chlorophyll content in beet leaves under 0–72 h drought stress (red line). (**G**–**L**) The PRO, MDA, POD, SOD, CAT, and chlorophyll content in beet leaves within 0–6 d rehydration (blue line)/CK (black line). The error line represents the standard deviation of three biological repetitions. The data of the CK group (without drought treatment) and the rehydration group at different time points are marked by the letter method of significant difference. There is no significant difference among the same letter groups (*p* > 0.05), but there are significant differences among different letter groups (*p* < 0.05). *, **, and *** represent significant differences at *p* < 0.05, 0.01, and 0.001 levels, respectively, between the CK group and the rehydration group at common moments.

Changes in physiological indicators of sugar beets under drought stress were assessed by measuring the levels of PRO, MDA, POD activity, SOD activity, CAT activity, and chlorophyll content in the leaves of sugar beet seedlings. These measurements were conducted at seven distinct time points during drought stress treatment and at four time points following rehydration treatment (Figure 2).

Drought treatment had significant effects on the physiological indexes of plant seedlings. The content of PRO fluctuated slightly during 0~12 h, increased continuously after 12 h, and reached the maximum value at 72 h, suggesting that plants respond to adversity by synthesizing more PRO. The MDA rose rapidly after 6–12 h of decline and peaked at 24 h, reflecting increased oxidative stress caused by drought. POD content rose in 0–12 h and declined in 12–24 h. The SOD increased from 0 to 24 h and peaked at 24 h, then decreased at 48 h and increased again at 48 to 72 h. The CAT increased from 0 to 12 h, peaked at 12 h, decreased from 12 to 48 h, and increased again at 48 to 72 h. In a different way, chlorophyll content began to decrease continuously from 0 to 24 h to the lowest and then gradually recovered during 24–72 h and rose to the highest level at 72 h. These changes indicated that plants had some drought-tolerant adaptation mechanisms.

During the plants rewatering process after drought stress, the physiological indexes changed significantly at different time points, and all physiological indexes were higher than those in the CK group. PRO content decreased in 0–2 d then slowly increased, indicating that plants gradually adapt to the environment after rewatering. The content of MDA decreased in 0–4 d, reached the minimum value on the 4th day, and increased slightly in 4–6 d. POD content increased in 0–2 d and nearly stayed constant within 2–6 d, indicating that the plants maintain antioxidant capacity after rehydration. The content of SOD increased continuously in 0–6 d. The CAT decreased rapidly in 0–2 d, kept constant in 2–4 d, and decreased slightly in 4–6 d. The content of chlorophyll decreased continuously in 0–6 d, which indicated that the photosynthesis of plants was affected after rewatering. These changes indicated that rewatering treatment promoted plant recovery to a certain extent, but it still took time to fully adapt and repair the physiological damages caused by drought.

### 2.3. Bioinformatics Analysis of BvSPL6/7/9 Genes

Three targets of the beet *miR156* gene, specifically the genes *BvSPL6*, *BvSPL7*, and *BvSPL9*, were identified through database predictions. These genes are located on different chromosomes. The analysis of the physicochemical properties of the amino acids associated with three beet transcription factors (BvSPL6/7/9) is presented in Table 1 below.

**Figure 3:**
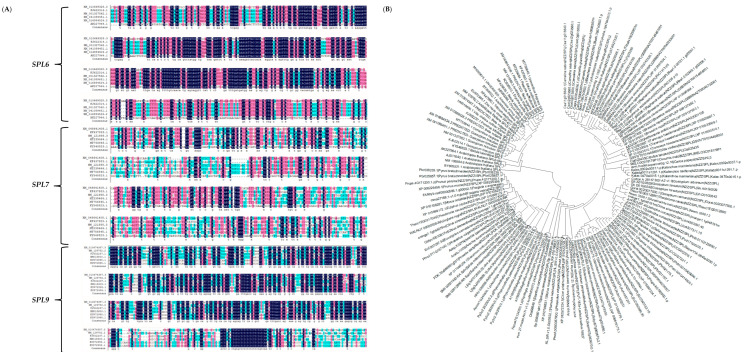
Sequence alignment analysis of *BvSPL6/7/9* genes in sugar beets. (**A**) Multiple alignment of amino acid sequences encoded by *miR156* target genes *BvSPL6/7/9* and other plant *miR156* target genes. (**B**) Phylogenetic tree analysis of *BvmiR156* target gene and other plants *miR156* target gene family.

DNAMAN was employed to compare the amino acid sequences of BvSPL6, BvSPL7, and BvSPL9, revealing a high degree of homology among them. Each domain exhibited strong conservation, with sequence similarity reaching significant levels, including numerous homologous sites with 100% identity, as illustrated in Figure 3A. To further analyze the homology of the target genes, a BLAST functional analysis was conducted on the NCBI website, focusing on the nucleotide sequences of the sugar beet *BvSPL6/7/9* genes. Several dicotyledonous plants with the highest homology, including *Arabidopsis*, apple, grape, cotton, and rice, were selected for comparative analysis of their *miR156* target genes. A phylogenetic tree was constructed using MEGA7 software. The results indicated that the evolutionary relationship between *BvSPL6* and cotton (XM041089451.1) was the closest; *BvSPL7* was most closely related to peony (MT239464.1); and *BvSPL9* showed the closest relationship to Arabidopsis (NM129782.2). Additionally, the evolutionary distances among the *BvmiR156* target genes were relatively short, as depicted in Figure 3B.

The protein primary sequence analysis revealed that the transcription factors BvSPL6, BvSPL7, and BvSPL9 had molecular formulas of C_2315_H_3655_N_687_O_762_S_24_, C_3885_H_6054_N_1076_O_1204_S_56_, and C_1701_H_2641_N_523_O_558_S_17_, respectively. BvSPL6 had a molecular weight of 54073.07 Da and consists of 487 amino acids, with polar amino acids (Arg and Lys) accounting for 10.6% and nonpolar amino acids (Asp and Glu) accounting for 10.5%. It was predicted that the isoelectric point (pI), instability index, and aliphatic index were 7.55, 58.26, and 64.62, respectively, indicating its classification as an unstable protein. BvSPL7, with a molecular weight of 88894.65 Da and 791 amino acids, had polar amino acids comprising 10.9% and nonpolar amino acids comprising 13.9%. The predicted pI, instability index, and aliphatic index were 5.52, 45.09, and 72.55, respectively, categorizing it as an unstable protein. BvSPL9, weighing 39890.87 Da and containing 376 amino acids, had polar amino acids at 9.3% and nonpolar amino acids at 7.7%. The predicted pI, instability index, and aliphatic index were 8.83, 58.26, and 53.91, respectively, indicating its classification as an unstable protein as well. These findings suggested that all three transcription factors were unstable hydrophilic proteins, albeit with some differences in amino acid composition and physicochemical properties.

**Figure 4:**
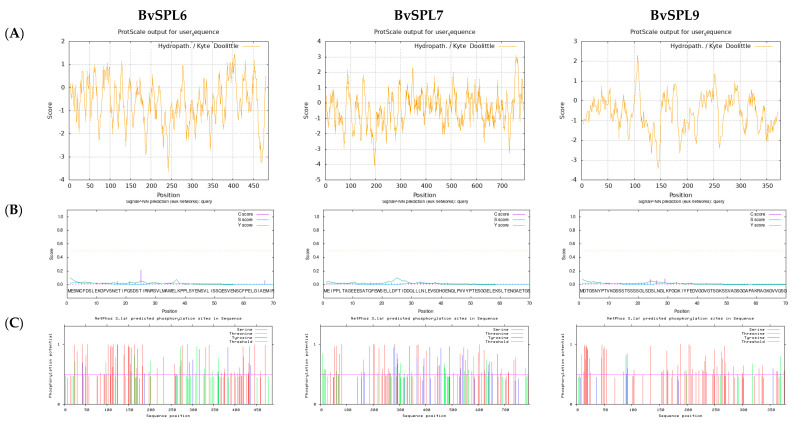
Prediction of hydrophobicity, signal peptide and amino acid processing modification. (**A**) Hydrophobic analysis, (**B**) Signaling peptides prediction, and (**C**) Predicted phosphorylation sites for transcription factors BvSPL6/7/9, respectively.

Based on the hydrophobicity analysis, the transcription factors BvSPL6, BvSPL7, and BvSPL9 were predicted to be hydrophilic proteins, as most of their sequences score negative values (Figure 4A). Cell-PLoc 2.0 prediction results showed that BvSPL6, BvSPL7, and BvSPL9 transcription factors may act within the nucleus, with no signal peptides detected, indicating that they are non-secreted proteins. According to Cell-PLoc 2.0 predictions, BvSPL6 and BvSPL9 were tentatively identified to function within the nucleus, and BvSPL7 was identified to function within the cytoplasm and nucleus. Signal peptide predictions indicated that BvSPL6/7/9 transcription factors had a signal peptide probability of zero, suggesting that they did not contain signal peptides and were non-secretory proteins (Figure 4B).

The phosphorylation analysis using NetPhos 2.0 Serve unveiled notable phosphorylation alterations within the BvSPL6, BvSPL7, and BvSPL9 protein sequences, as shown in Figure 4C. BvSPL6 displayed a substantial count of phosphorylated serine (53), threonine (12), and tyrosine (10) residues. Similarly, BvSPL7 exhibited heightened levels of serine phosphorylation (47), hinting at potential involvement in signaling cascades and cellular regulatory mechanisms. Moreover, BvSPL7 showcased significant phosphorylation on threonine (20) and tyrosine (16) residues. BvSPL9 also demonstrated significant phosphorylation, notably with up to 50 serine residues, implying a probable function in signaling pathways and cellular regulation. Additionally, BvSPL9 presented phosphorylation on threonine (7) and tyrosine (2) residues.

**Figure 5:**
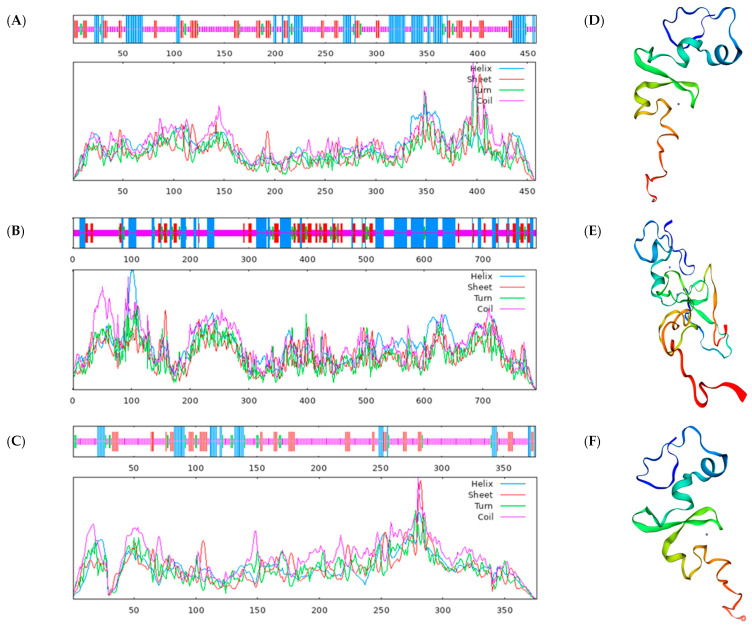
Prediction of protein secondary and tertiary structures. Prediction of the secondary structure of the three transcription factors (**A**) BvSPL6, (**B**) BvSPL7, and (**C**) BvSPL9. Prediction of the tertiary structure of the three transcription factors (**D**) BvSPL6, (**E**) BvSPL7, and (**F**) BvSPL9. Blue represents α-spiral structure, red represents β-sheet, green represents β-turn and purple represents random coil.

Utilizing SOPMA for online prediction of protein secondary structures, we acquired the secondary structures of BvSPL6/7/9 transcription factors. In the case of the BvSPL6, 24.02% of the protein secondary structure comprised α-helical structures, with 17.69% extended chains, 4.37% β-sheet structures, and 53.93% irregular coils. For the BvSPL7, 30.09% of the protein secondary structure consisted of α-helices, with 16.06% extended chains, 4.93% β-sheets, and 48.93% irregular coils. Regarding the BvSPL9, 11.70% of the protein secondary structure was α-helical, with 14.63% extended chains, 5.59% β-sheets, and 68.09% irregular coils (Figure 5A–C). Employing the Swiss Model website for tertiary structure prediction (Figure 5D–F), we observed that the structure of sugar beet BvSPL6 protein exhibited the highest similarity to *Arabidopsis* BvSPL6 protein, at 63.64%. The structure of sugar beet BvSPL7 protein displayed the highest resemblance to *Arabidopsis* BvSPL7 protein, at 84.38%. Similarly, the structure of sugar beet BvSPL9 protein demonstrated the highest similarity to *Arabidopsis* SPL9 protein, at 67.14%.

### 2.4. Cloning of BvSPL6/7/9 Genes in Beet

**Figure 6:**
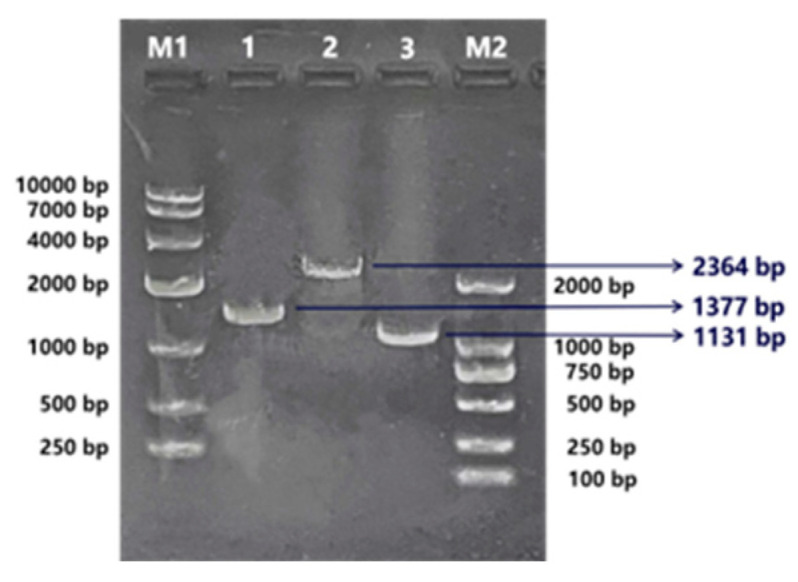
Electrophoresis results of PCR products of cloned *BvSPL6/7/9* genes. M1 is markerDL10000, M2 is markerDL2000, and 1–3 lanes are samples of cloned results. 1: *BvSPL6*, 2: *BvSPL7*, 3: *BvSPL9*.

The PCR electrophoresis results of the bacterial solution post single colony amplification and cultivation are depicted in Figure 6. In lane 1, a band of approximately 1377 bp emerged, while a strip of around 2364 bp was observed in lane 2. Additionally, a strip of roughly 1131 bp appeared in lane 3. The target bands corresponding to *BvSPL6*, *BvSPL7*, and *BvSPL9* were successfully amplified in bacterial solutions 1–3. This validated the successful transformation of bacterial liquid in solutions 1–3, indicating the successful cloning of the three genes *BvSPL6/7/9*, ready for sequencing.

The gene sequencing analysis revealed that the *BvSPL6* gene in sugar beets has a total length of 2745 bp, which included a coding region of 1377 bp that encoded a protein of 487 amino acids. Similarly, the *BvSPL7* gene exhibited a total length of 3054 bp, with a coding region spanning 2364 bp, resulting in a protein composed of 791 amino acids. In contrast, the *BvSPL9* gene was 1587 bp in length, contained a coding region of 1131 bp that encoded 376 amino acids.

### 2.5. Construction of BvSPL6/7/9 Expression Vectors

**Figure 7:**
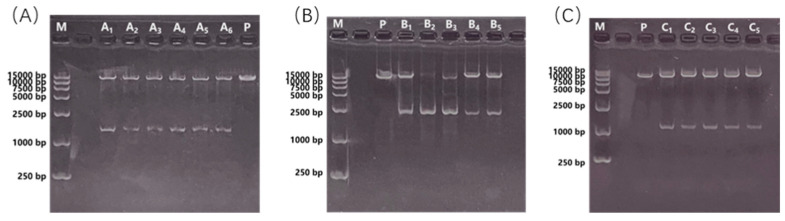
Double enzyme digestion electrophoresis results of *BvSPL6/7/9* gene expression vectors. M is 15,000 bp Maker, P is an empty plasmid pCAMBIA3301 as a control, A_1_–A_6_, B_1_–B_5_, and C_1_–C_5_ are double enzyme digestion results. (**A**) *BvSPL6*, (**B**) *BvSPL7*, (**C**) *BvSPL9*.

The cleavage sites flanking the *BvSPL6/7* gene products are *BamH I* and *Xba I*, respectively. For the *BvSPL9* gene product, the cleavage sites at both ends are *EcoR I* and *Xba I*, which are compatible with the restriction sites of the expression vector. Double enzyme digestion was performed on the sequencing vectors T-*BvSPL6*, T-*BvSPL7*, and T-*BvSPL9*, as well as on the plant overexpression vector pCAMBIA3301. Following electrophoresis, the target bands were recovered, and the vectors were transformed into *Escherichia coli* after matching and ligating according to their molar concentrations. Positive recombinant bacteria were obtained after screening and cultivation, and plasmid extraction was conducted for double enzyme digestion to confirm correctness. Following identification through double restriction enzyme digestion, positive recombinants were screened. The presence of two bands on the electrophoresis gel indicated successful ligation, leading to subsequent sequencing verification. The electrophoresis results confirmed that the *BvSPL6/7/9* vectors have been successfully recombined (Figure 7).

**Figure 8:**
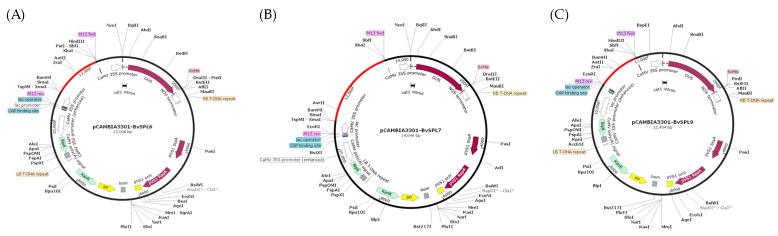
Map of *BvSPL6/7/9* gene expression vectors (**A**) pCAMBIA3301-*BvSPL6*, (**B**) pCAMBIA3301-*BvSPL7*, and (**C**) pCAMBIA3301-*BvSPL9*.

The sizes of the two restriction enzyme digestion products were consistent with expectations: the plasmid length was 11,320 bp, while the gene fragment lengths were as follows: *BvSPL6* with 1377 bp, *BvSPL7* with 2364 bp, and *BvSPL9* with 1131 bp. These results confirmed that all constructs were positive recombinants. The samples were subsequently sent for sequencing, and comparative analysis verified that the sequences were completed, indicating their suitability for plant transformation experiments. Figure 8 presents a schematic diagram of the gene overexpression vector construction.

### 2.6. Agrobacterium Mediated Transformation and Expression Analysis of BvSPL6/7/9 Genes in Sugar Beets

**Figure 9:**
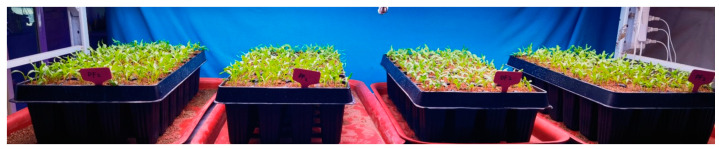
Sugar beet seedlings before transient infection by *Agrobacterium tumefaciens*.

The constructed expression vectors pCAMBIA3301-*BvSPL6*, pCAMBIA3301-*BvSPL7*, and pCAMBIA3301-*BvSPL9* were transformed into *Agrobacterium tumefaciens*, which were then used to infect beet cotyledons. Sugar beet seedlings were cultivated until the first pair of true leaves developed and the cotyledon leaves reached a length of 2 cm or more, at which point they were ready for infection, as shown in Figure 9. A 5 mL sterile syringe was filled with the bacterial solution for injection and inoculation. The lower epidermis of the cotyledon leaves was gently tapped with a needle, and the bacterial solution was slowly injected into the leaf tissue until it was visibly infiltrated by the solution.

**Figure 10:**
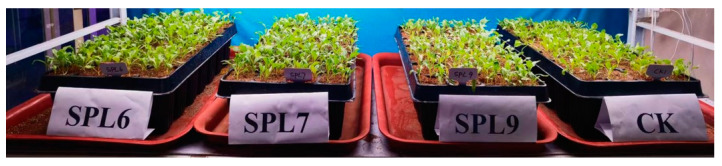
Sugar beet seedlings infected by *Agrobacterium tumefaciens* for 7 days.

Following the infection, the entire beet seedlings exhibited a wilting state characterized by drooping leaves. The plants were subsequently transferred to a low-light, well-ventilated area for further cultivation. After approximately 7 days, the plants resumed their normal growth form, as depicted in Figure 10.

**Figure 11:**
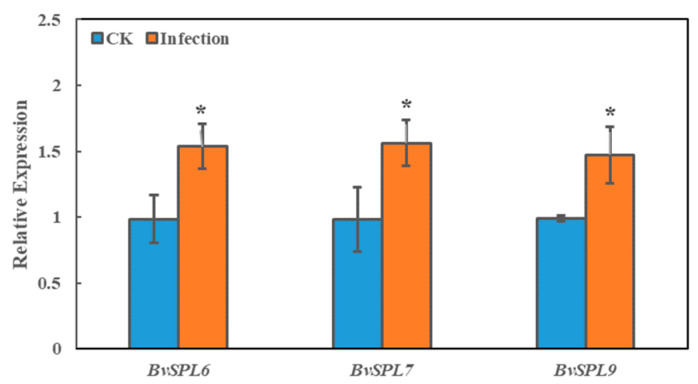
Overexpression of *BvSPL6/7/9* genes in leaves of transgenic sugar beet seedlings. The error line and * represent the standard deviation of the three biological repetitions and a significant difference at the *p* < 0.05 level, respectively.

When the plant reached the required size, the beet leaves were frozen in liquid nitrogen and stored in a −80 °C refrigerator for later use. The leaf parts of transgenic sugar beet seedlings and CK sugar beet seedlings were selected, the leaf stems and larger leaf veins were removed, and total RNA was extracted and then and reverse transcribed into cDNA for fluorescence quantitative analysis. As shown in Figure 11, the relative expression levels of *BvSPL6*, *BvSPL7*, and *BvSPL9* genes were significantly higher in three overexpressed transgenic sugar beet plants than those in the CK group, indicating that the *BvSPL6/7/9* genes were transferred into the sugar beet seedlings and detected. Expression analysis in transgenic sugar beets indicated that the *BvSPL6/7/9* genes had been successfully transformed into sugar beet seedlings and expressed. This result provided a preliminary basis for subsequent research on drought resistance of sugar beets.

**Figure 12:**
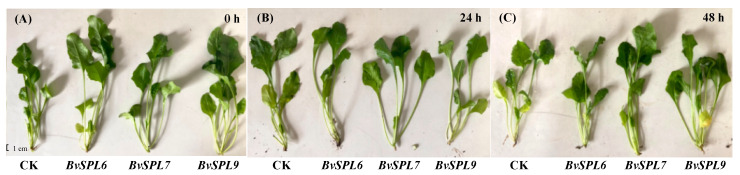
Phenotypes of transgenic sugar beet seedlings under drought stress treatment. (**A**–**C**) represent drought treatment with 15% PEG6000 for 0 h, 24 h, and 48 h, respectively. The four sugar beet seedlings from left to right corresponded to CK seedling, *BvSPL6*, *BvSPL7*, and *BvSPL9* overexpressing seedlings, respectively.

When the indoor beet seedlings reached the two to three pairs of true leaves stage, the DF-2 strain seedlings were subjected to drought stress by treatment with 15% PEG6000 for durations of 0 h, 2 h, 6 h, 12 h, 24 h, 48 h, and 72 h, respectively, with a CK group established for comparison. The seedling phenotypes of the DF-2 strain of sugar beets under drought stress are illustrated in Figure 12. At the 0 h drought treatment, there were no significant differences observed among the CK group seedlings and three group *BvSPL6/7/9* overexpressing seedlings, as shown in Figure 12A. Water loss in the CK group began after 24 h of drought treatment, as depicted in Figure 12B. The dewatering effects observed in both *BvSPL6*/7 overexpressing seedlings after 48 h of drought treatment were presented in Figure 12C. These results indicated that three *BvSPL6/7/9* genes were involved in the response to drought stress. It could be concluded that the *BvSPL6/7/9* genes can respond to oxidative stress by upregulating their expression, thereby delaying the onset of water loss.

## 3. Discussion

By analyzing the CDS of the *BvSPL6*, *BvSPL7*, and *BvSPL9* genes in beet, we constructed their overexpression vectors. Comparative bioinformatics analysis revealed a higher degree of homology between these genes and their Arabidopsis counterparts. The secondary structure of the proteins is characterized by irregular coiling, and the hydrophobic regions contain a highly conserved SBP domain, indicating their classification within the *SPL* gene family. It has been established that SPL transcription factors play a crucial role in regulating plant development and stress responses. *SPLs* are significant inducers of plant stress resistance, mediating the plant’s ability to withstand various stresses, including drought and low temperatures [20]. The first *microRNA* identified to regulate the SPL transcription factor, *miR156*, was discovered in 1997 [21]. The *miR156*/SPL regulatory module is crucial for various developmental processes, including embryogenesis, transitions between developmental stages, flowering time, and morphogenesis, as well as playing a significant role in plant responses to stress [20,22,23,24]. Our findings indicated that the expression of the *BvSPL6* and *BvSPL9* genes in leaves is upregulated during the mid-stage of drought stress, while their expression is downregulated during the early and late stages of drought stress. In contrast, the expression of the *BvSPL7* gene progressively increases with the duration of drought stress. These results suggested that *BvSPL6*, *BvSPL7*, and *BvSPL9* may be involved in the regulation of drought resistance-related genes in beets, contributing to the plant’s response to drought stress. Based on the relative expression trends of the three genes, we hypothesized that the *BvSPL7* gene was regulated by activation. The expression patterns of *BvSPL6* and *BvSPL9* exhibited similar trends, suggesting a relatedness in their expression and regulatory networks; however, the patterns of increase and decrease were relatively complex. This complexity implied that *miR156* may not be the sole regulator during the growth of beets. For instance, *miR529*a has been shown to control the growth and development of rice by regulating distinct *SPL* target genes at various developmental stages [25]. Additionally, the *ARF3* gene played a regulatory role through direct interaction with the *SPL* promoter, thereby limiting SPL expression in microspore mother cells. This regulation prevented excessive and potentially harmful levels of SPL expression, ensuring the accurate progression of early anther morphogenesis [26]. Furthermore, the overexpression of *miSPL13* not only promoted early flowering in transgenic Arabidopsis, leading to increased expression levels of AtAP1, AtSOC1, and AtFUL, but also significantly enhanced tolerance to drought, ABA, and GA3, while exhibiting sensitivity to Pro-Ca treatment [27]. Plant SPL transcription factors were capable of binding to DNA to regulate gene expression and were also subject to regulation by other genes, thereby influencing downstream gene activity and forming a complex gene regulatory network. In summary, the relative expression trends of the three genes suggest that *BvSPL7* is regulated by activation, while the similar expression patterns of *BvSPL6* and *BvSPL9* indicate their interconnectedness within the expression and gene regulatory network, albeit with complex modulation. It is plausible that *miR156* is not the only regulatory factor in the response of sugar beet seedlings to drought treatment or that *miR156* may exert both inhibitory and degradation effects on its target genes.

Following rehydration treatment, the expression levels of *BvSPL6*, *BvSPL7*, and *BvSPL9* exhibited varying degrees of change. Specifically, the expression levels of *BvSPL6* and *BvSPL9* in the leaves of sugar beet seedlings displayed a similar trend from 0 to 72 h, characterized by an initial decrease followed by an increase. In contrast, the expression level of *BvSPL7* increased initially and then decreased. Compared to the unstressed CK group, the expression of *BvSPL6* and *BvSPL9* was generally downregulated during the period of 0 d to 6 d, while *BvSPL7* was predominantly upregulated during this period. A comparison between the CK group and the rehydration treatment group revealed that the expression levels of *BvSPL6* and *BvSPL9* were lower in the treatment group than in the unstressed CK group within the 0 d to 6 d timeframe. Notably, during the 4 d rehydration period, the expression of *BvSPL6* in the treatment group was significantly lower than that in the CK group, indicating a marked reduction in the relative expression of *BvSPL6* during the mid-stage of rehydration. Similarly, the expression of *BvSPL9* in the treatment group was significantly lower than that in the CK group throughout the rehydration period. Conversely, *BvSPL7* exhibited significantly higher expression levels compared to the CK group within the 0 d to 6 d period. These findings suggested that during the 6-day rehydration recovery treatment, *BvSPL6* and *BvSPL9* in sugar beet seedlings responded to rehydration by downregulating their expression, while *BvSPL7* underwent rehydration recovery through upregulation. This indicated that the degree of irreversible damage to the plants during drought treatment was relatively low, allowing them to restore normal growth through rehydration.

In this study, drought stress experiments were conducted on the DF-2 sugar beet strain, which was subjected to drought stress treatment at the stage of two to three pieces of true leaves using a 15% PEG6000 solution for various durations (0, 2, 6, 12, 24, 48, and 72 h) followed by rehydration for 0, 2, 4, and 6 d. Quantitative analyses were performed on sugar beet leaves at different treatment time points to investigate changes in the expression levels of the three genes under drought stress and rehydration conditions. Bioinformatics techniques were employed to analyze the *BvSPL6*, *BvSPL7*, and *BvSPL9* genes, providing preliminary insights into the stress-resistance mechanisms associated with these genes. Additionally, it was observed that tomatoes also exhibited gradual recovery after drought stress following 96~264 h of rewatering [25].

The *BvSPL6/7/9* genes play a critical role in regulating proline content in sugar beet leaves under conditions of drought stress and subsequent rehydration recovery, indicating their significant involvement in the molecular mechanisms underlying osmotic stress responses in sugar beet cells. Furthermore, these genes are implicated in the regulation of chlorophyll content during drought stress and rehydration recovery, suggesting that they are essential for the molecular regulation of photosynthesis in sugar beet. Additionally, *BvSPL6/7/9* genes modulate the activities of SOD, POD, and CAT in leaves subjected to drought stress and rehydration recovery, highlighting their importance in the oxidative stress response mechanisms of sugar beet cells. The regulation of MDA content by *BvSPL6/7/9* genes under drought stress and rehydration recovery further suggests their critical role in maintaining cellular membrane integrity. Sugar beets utilize MDA to mitigate membrane lipid peroxidation, thus preserving membrane functionality and enhancing drought tolerance. Collectively, these findings imply that the regulatory mechanisms governing drought resistance and rehydration recovery in sugar beets in response to oxidative stress are closely linked to the significant upregulated expression of *BvSPL6/7/9* genes under drought conditions, alongside the notable downregulated expression of *BvSPL6/9* genes during rehydration stress.

Plant stress tolerance is a multifaceted process that mitigates the detrimental effects of adverse environmental conditions through protein synthesis and is intricately regulated by various genes. In recent years, the modified *miR156* target node has emerged as a promising tool in crop adaptation to abiotic stress, thereby enhancing the potential for genetic engineering in plant resilience. The genetic modification of plants via the *miR156*-SPLs-DFR pathway effectively coordinates plant development with abiotic stress tolerance [28]. Moreover, numerous studies have demonstrated that endogenous signaling molecules, including gibberellin, ABA, and JA, regulated the DELLA proteins associated with *SPL*, thereby influencing plant stress responses [13,29,30]. The significance of plant immunity has been underscored by recent research identifying the roles of *miR156* and *SPL* in mediating responses to biotic stress. Although *miR156* has been utilized in molecular crop breeding for several years, a more comprehensive understanding of its gene regulatory network is necessary to elucidate its role in enhancing crop quality and disease resistance. Notably, *miR156* and certain *SPL* genes can be modulated by secondary metabolites to bolster plant resilience, such as the enhancement of anthocyanin content [28].

*MiR156* and its target SPL transcription factor family play an important role in plant response to stress. However, there are still many biological problems to be solved. First, how the *miR156*/SPL pathway interacts with other signaling pathways, such as hormonal and environmental signals, under biological stress remains to be further investigated. In addition, further identification and validation of other *miR156*-regulated target genes will be helpful for revealing the breadth and diversity of this pathway. The use of gene editing techniques to precisely regulate the expression of *miR156* or SPL may provide a new strategy for enhancing crop stress resistance. By combining genomic, transcriptomic, and metabolomic analyses, we can fully understand the complex roles of *miR156*/SPL in plant growth and stress response. These studies will lay a solid foundation for future applications in plant biology and agriculture.

## 4. Materials and Methods

### 4.1. Cultivation and Stress Treatment of Beet Seedlings

In this study, the Harbin Institute of Technology Sugar Beet Line DF-2 was selected by the sugar beet breeding research group for investigation. Screening results indicated that a 15% PEG6000 solution exhibited a superior inhibitory effect on drought stress. Sugar beet seeds were initially soaked in tap water for 12 h, followed by disinfection with 75% ethanol for 1 to 2 min. The seeds were then rinsed again with tap water and air-dried in the shade for 2 to 3 h. Subsequently, the seeds were sown in a sterile mixture of vermiculite and nutrient soil and cultured at a temperature of 24 °C with a photoperiod of 16 h light and 8 h dark. Once the seedlings developed cotyledons, 1 to 2 seedlings were retained in each pot. The sugar beet seedlings were subjected to 15% PEG6000 treatment after developing 2 to 3 pairs of true leaves, with exposure durations of 0, 2, 6, 12, 24, 48, and 72 h. Concurrently, non-drought control plants were established for comparison. Following the drought stress treatment, samples were collected at 0, 2, 4, and 6 d post-treatment. Leaf samples, each weighing 0.2 g, were wrapped in aluminum foil, rapidly frozen in liquid nitrogen, and subsequently stored in an −80 °C ultra-low temperature freezer.

### 4.2. Bioinformatics Analysis of Drought-Tolerant Genes in Sugar Beets

Compared with model plants, the entire genome sequence of sugar beets has not yet been fully disclosed. Therefore, this study used homologous alignment to predict the function of its target genes and chose a more relaxed alignment parameter to find more potential target genes. For the analysis of base sequence information of the target gene, information was obtained from the sugar beet gene database and compared to NCBI to obtain sequence number, base sequence, and fragment length information, while obtaining its chromosome localization. The promoter location was found through the SoftBerry website, base preferences were analyzed using MEGA7.0 software, and a phylogenetic tree was constructed. For the analysis of protein primary structures, the protein primary amino acid sequence of the target gene can be obtained on NCBI and submitted online to the ProParam website to analyze the physical and chemical properties of the target gene, including molecular weight, isoelectric point, amino acid composition, total average hydrophilicity, etc. A detailed analysis of the hydrophilicity and hydrophobicity of the amino acid sequence of the target gene can be performed using ProScale, which can reveal the hydrophilicity of each part of the sequence, including which segment is a high hydrophilicity region. For the analysis of protein secondary structure, the amino end sequence of the target gene is also required. The amino acid sequence is submitted to the PredictProtein website, which can predict which segments the spiral chain, extension chain, and irregular curl chain are, respectively. At the same time, the proportion of various amino acids is analyzed to facilitate a deeper understanding of the physical and chemical properties of the protein. For the analysis of protein tertiary structure, the amino acid sequence can be submitted to Swiss Model, which can predict the three-dimensional structure of the target protein.

A search was conducted on miRBase for *miR156* (www.miRBase.org/index.shtml (accessed on 20 October 2023)). The psRNA website (plantgrn.noble.org/psrnatarget/ (accessed on 20 October 2023)) was used to predict *miRNA* targets in sugar beets. Target gene function was predicted by homology comparison. The sequence number, base sequence, and fragment length of the target gene were obtained from the beet gene database and compared with NCBI. The chromosome position of the target gene was obtained through the SoftBerry website. Mega7.0 software was used to analyze the basic preferences and build the phylogenetic tree. The amino acid sequences of the target genes were obtained from NCBI and submitted to the PROPARAM website for physical chemistry analysis and hydrophobicity analysis using ProScale. Finally, the information was submitted to Swiss Model for predicting the three-level structure.

### 4.3. Physiological Experiments

At 0, 2, 6, 12, 24, 48, and 72 h of drought stress treatment, rehydration time was 0, 2, 4, and 6 d, and the corresponding CK group of the physiological experiment was used in Suzhou Greg, biological technology Co., Ltd. (Suzhou, China). The contents of PRO assay kits, MDA content determination kit, SOD kits, POD kit, and CAT kits were used to determine the corresponding physiological indexes.

### 4.4. Quantitative Analysis of Target Genes under Drought Stress

A Takara MiniBEST Plant RNA Extraction Kit (Suzhou, China) was used. Total RNA was detected by 1.5% agarose gel electrophoresis. RNA was reverse transcribed using the PRIMESCRIPT^TM^ II 1st Strand cDNA Synthesis Kit (Suzhou, China), following the instructions. qRT-PCR primers were designed with Premier 5 software, and ICDH was used as the reference gene and synthesized by Rui Boxingke Co. Ltd. (Suzhou, China). The instrument used for the qRT-PCR fluorescence quantification experiments of the genes was a CFX96 fluorescence quantitative PCR instrument (USA) with a fluorescent dye from the ITAQ^TM^ Universal SYBR^®^ Green Supermix Kit from BIO-RAD (USA). Each sample was repeated three times according to the kit instructions. The primer names and sequences for qRT-PCR are shown in Table 2.

### 4.5. Cloning of Target Genes

The target gene was cloned using a sugar beet DF-2 strain treated with 15% PEG6000 drought stress for 72 h. The target gene sequence of the model organism Arabidopsis thaliana was queried on the Internet, and then the homology was compared to the same sequence in the beet reference genome. The base sequence was downloaded from NCBI and imported into DNAMAN 6.0, and the restriction sites in the fragment were then analyzed. After excluding the restriction sites, two suitable insertion sites on the expression vector were found and added to both ends of the target gene primer. Primer Premier 5 was used to design primers for this sequence. Using primers, templates, and high-fidelity enzyme KOD Plus to amplify the target gene, the reaction system 50 μL PMD using the TAKARA™ The 18-T Vector Cloning Kit (Suzhou, China) connected the recovered target genes to the T vector overnight at 16 °C. The connected plasmid was transformed into competent cells of *Escherichia coli* and expanded in *Escherichia coli* with the proliferation of *Escherichia coli*. Further confirmation of the positive clone bacterial solution was obtained through screening, screening out the positive clone bacterial solution. The positive clone bacterial solution was taken. The names and sequences of primers for gene cloning are shown in Table 3.

### 4.6. Statistical Analysis

All experiments were performed in three biological and three analytical replicates. Data were subjected to analysis of variance using SPSS data analysis software version 20.0 (SPSS 20.0). The results were presented as average means and standard deviations. Statistically significant differences at *p* < 0.05 are discussed. Moreover, a professional drawing software (Origin 9.5) was used for data plotting.

## 5. Conclusions

In this study, *BvSPL6*, *BvSPL7*, and *BvSPL9* genes in sugar beets were identified and analyzed, and their expression under drought stress was discussed. The expressions of *BvSPL6/9* genes were upregulated in the middle stage of drought stress and downregulated in the early and late stages of drought stress. The expression of *BvSPL7* was gradually upregulated with the increase in drought stress duration. These results indicated that the regulation of *BvSPL6/7/9* genes was closely related to drought resistance in sugar beets. At the same time, rewatering treatment promoted plant recovery to some extent, but it took some time to fully adapt to and repair the physiological damage caused by drought. In addition, the *BvSPL6*, *BvSPL7*, and *BvSPL9* gene expression vectors were successfully transferred into sugar beet seedlings by *Agrobacterium tumefaciens* transformation. *BvSPL6/7/9* genes were positively regulated in *SPL*-overexpressing seedlings after drought treatment. In conclusion, *BvSPL6*, *BvSPL7*, and *BvSPL9* genes play an important role in sugar beet drought resistance and have potential application value.

## Figures and Tables

**Table 1:** Analysis of Amino Acid Physicochemical Properties of Transcription Factors BvSPL6/7/9 in Sugar Beets.

Transcription Factor Name	BvSPL6	BvSPL7	BvSPL9
Gene search number	XM_010669328.3	XM_048641408.1	XM_010674387.3
Chromosome position	1	3	3
*BvSPL*-gene length (bp)	2745	3054	1587
CDS area length (bp)	1377	2364	1131
Number of encoded amino acids	487	791	376
Number of exons	10	24	12
Number of introns	12	3	4
Isoelectric point	7.55	5.52	8.83
Protein molecular weight (Da)	54,073.07	88,894.65	39,890.87
Instability index	58.26	45.09	58.26
Aliphatic index	64.62	72.55	53.91
GRAVY	−0.638	−0.398	−0.610
Max missed cleavages	42	65	45
Number of promoters	635	916	513
Protein kinases	CKI, PKC, cdc2, unsp	CKI, PKC, cdc2, unsp	PKC, CKI, unsp

**Table 2:** Primer names and sequences used in qRT-PCR.

Gene	Forward Primer (5′→3′)	Reverse Primer (5′→3′)
*BvICDH*-F	CACACCAGATGAAGGCCGT	CCCTGAAGACCGTGCCAT
*BvSPL6*-1F	CGGTAGTGCTGGTGTTGGAGATATG	GGCTGCTCTAGTTGAATCAGAATTAGGT
*BvSPL7*-1F	ACTTCAGTTGTGCTTGATGGTCAGAG	ACTTCCTTCGCCGTCTATTGTTGTG
*BvSPL9*-1F	GTGGTGGATTCTTATTGGACTTCTCTT	CACATAATGGACCTGGACTGACTTC

**Table 3:** Primer names and sequences used for gene cloning.

Gene	Forward Primer (5′→3′)	Reverse Primer (5′→3′)
*BvSPL6*-2F	CGGGATCCATGGATTCTTGGAAATATATGTCAG	GCTCTAGATTAACCTGCTTCATTTTCAGTTGTATGCC
*BvSPL7*-2F	CGGGATCCATGGAAATACCACC	GCTCTAGACTATGAAGAATTGTCAAACACG
*BvSPL9*-2F	CGGAATTCATGGATACGGGTTCA	GCTCTAGATCAGAGTGACCAGTCCACAT

## Data Availability

Data are contained within the article.

## References

[1] Zou C., Liu D., Wu P., Wang Y., Gai Z., Liu L., Yang F., Li C., Guo G. (2020). Transcriptome analysis of sugarbeet (*Beta vulgaris* L.) in response to alkaline stress. Plant Mol. Biol..

[2] Zhang Q., Yao Y., Li Y., Huang J., Ma Z., Wang Z., Wang S., Wang Y., Zhang Y. (2020). Causes and Changes of Drought in China: Research Progress and Prospects. J. Meteorol. Res..

[3] Yu X., Zhang L., Zhou T., Zhang X. (2023). Long-term changes in the effect of drought stress on ecosystems across global drylands. Sci. China Earth Sci..

[4] He F., Long R., Wei C., Zhang Y., Li M., Kang J., Yang Q., Wang Z., Chen L. (2022). Genome-wide identification, phylogeny and expression analysis of the SPL gene family and its important role in salt stress in *Medicago sativa* L.. BMC Plant Biol..

[5] Wang H., Wang H. (2015). The miR156/SPL Module, a Regulatory Hub and Versatile Toolbox, Gears up Crops for Enhanced Agronomic Traits. Mol. Plant.

[6] Birkenbihl R.P., Jach G., Saedler H., Huijser P. (2005). Functional Dissection of the Plant-specific SBP-Domain: Overlap of the DNA-binding and Nuclear Localization Domains. J. Mol. Biol..

[7] Klein J., Saedler H., Huijser P. (1996). A new family of DNA binding proteins includes putative transcriptional regulators of the *Antirrhinum majus* floral meristem identity gene SQUAMOSA. Mol. General Genet. MGG.

[8] Stief A., Altmann S., Hoffmann K., Pant B.D., Scheible W., Bäurle I. (2014). Arabidopsis *miR156* Regulates Tolerance to Recurring Environmental Stress through SPL Transcription Factors. Plant Cell.

[9] Min X., Luo K., Liu W., Zhou K., Li J., Wei Z. (2022). Molecular Characterization of the *miR156/MsSPL* Model in Regulating the Compound Leaf Development and Abiotic Stress Response in Alfalfa. Genes.

[10] Wang Q., Sun A., Chen S., Chen L., Guo F. (2018). *SPL6* represses signalling outputs of ER stress in control of panicle cell death in rice. Nat. Plants.

[11] Yanzhi Y., Jianmei D., Yihan T., Chen H., Zheng K., Zhan L., Lei L. (2021). The Copper Responsive Transcription Factor SPL7 Represses Key Abscisic Acid Biosynthetic Genes to Balance Growth and Drought Tolerance. bioRxiv.

[12] Abdel-Ghany S.E., Pilon M. (2008). MicroRNA-mediated Systemic Down-regulation of Copper Protein Expression in Response to Low Copper Availability in Arabidopsis. J. Biol. Chem..

[13] Li S., Cheng Z., Li Z., Dong S., Yu X., Zhao P., Liao W., Yu X., Peng M. (2022). *MeSPL9* attenuates drought resistance by regulating JA signaling and protectant metabolite contents in cassava. Theor. Appl. Genet..

[14] Hanly A., Karagiannis J., Lu Q.S.M., Tian L., Hannoufa A. (2020). Characterization of the Role of *SPL9* in Drought Stress Tolerance in Medicago sativa. Int. J. Mol. Sci..

[15] Xue G., Wu W., Fan Y., Ma C., Xiong R., Bai Q., Yao X., Weng W., Cheng J., Ruan J. (2024). Genome-wide identification, evolution, and role of SPL gene family in beet (*Beta vulgaris* L.) under cold stress. BMC Genom..

[16] Islam M.J., Kim J.W., Begum M.K., Sohel M.A.T., Lim Y. (2020). Physiological and Biochemical Changes in Sugar Beet Seedlings to Confer Stress Adaptability under Drought Condition. Plants.

[17] Hussein H.A., Mekki B.B., El-Sadek M.E.A., El Lateef E.E. (2019). Effect of L-Ornithine application on improving drought tolerance in sugarbeet plants. Heliyon.

[18] Wedeking R., Maucourt M., Deborde C., Moing A., Gibon Y., Goldbach H.E., Wimmer M.A. (2018). 1H-NMR metabolomic profiling reveals a distinct metabolic recovery response in shoots and roots of temporarily drought-stressed sugarbeets. PLoS ONE.

[19] Wedeking R., Mahlein A., Steiner U., Oerke E., Goldbach H.E., Wimmer M.A. (2017). Osmotic adjustment of young sugarbeets (*Beta vulgaris*) under progressive drought stress and subsequent rewatering assessed by metabolite analysis and infrared thermography. Funct. Plant Biol..

[20] Ma Y., Xue H., Zhang F., Jiang Q., Yang S., Yue P., Wang F., Zhang Y., Li L., He P. (2021). The *miR156/SPL* module regulates apple salt stress tolerance by activating MdWRKY100 expression. Plant Biotechnol. J..

[21] Cardon G.H., Höhmann S., Nettesheim K., Saedler H., Huijser P. (1997). Functional analysis of the Arabidopsis thaliana SBP-box gene *SPL3*: A novel gene involved in the floral transition. Plant J..

[22] Jerome Jeyakumar J.M., Ali A., Wang W., Thiruvengadam M. (2020). Characterizing the Role of the *miR156-SPL* Network in Plant Development and Stress Response. Plants.

[23] Long J., Liu C., Feng M., Liu Y., Wu X., Guo W. (2018). *miR156-SPL* modules regulate induction of somatic embryogenesis in citrus callus. J. Exp. Bot..

[24] Xie Y., Zhou Q., Zhao Y., Li Q., Liu Y., Ma M., Wang B., Shen R., Zheng Z., Wang H. (2020). FHY3 and FAR1 Integrate Light Signals with the *miR156-SPL* Module-Mediated Aging Pathway to Regulate *Arabidopsis* Flowering. Mol. Plant.

[25] Yan Y., Wei M., Li Y., Tao H., Wu H., Chen Z., Li C., Xu J. (2021). *MiR529a* controls plant height, tiller number, panicle architecture and grain size by regulating SPL target genes in rice (*Oryza sativa* L.). Plant Sci..

[26] Yang Q., Wang J., Zhang S., Zhan Y., Shen J., Chang F. (2023). ARF3-Mediated Regulation of SPL in Early Anther Morphogenesis: Maintaining Precise Spatial Distribution and Expression Level. Int. J. Mol. Sci..

[27] Zhu J., He X., Li Y., Zhang Y., Yu H., Xia L., Mo X., Zeng X., Yang J., Luo C. (2022). Genome-wide analysis of the mango SPL family and overexpression of *MiSPL13* confers early flowering and stress tolerance in transgenic *Arabidopsis*. Sci. Hortic.-Amst..

[28] Cui L., Shan J., Shi M., Gao J., Lin H. (2014). The *miR156-SPL9*-DFR pathway coordinates the relationship between development and abiotic stress tolerance in plants. Plant J..

[29] Chen G., Li J., Liu Y., Zhang Q., Gao Y., Fang K., Cao Q., Qin L., Xing Y. (2019). Roles of the GA-mediated SPL Gene Family and *miR156* in the Floral Development of Chinese Chestnut (*Castanea mollissima*). Int. J. Mol. Sci..

[30] Feyissa B.A., Amyot L., Nasrollahi V., Papadopoulos Y., Kohalmi S.E., Hannoufa A. (2021). Involvement of the *miR156/SPL* module in flooding response in *Medicago sativa*. Sci. Rep..

